# Redefining floristic zones in the Korean Peninsula using high‐resolution georeferenced specimen data and self‐organizing maps

**DOI:** 10.1002/ece3.6790

**Published:** 2020-09-24

**Authors:** Songhie Jung, Yong‐chan Cho

**Affiliations:** ^1^ Gwangneung Forest Conservation Center Korea National Arboretum Pocheon Korea

**Keywords:** biogeographical region, floristic zone, Korean Peninsula, regionalization, self‐organizing map, vascular plant

## Abstract

The use of biota to analyze the distribution pattern of biogeographic regions is essential to gain a better understanding of the ecological processes that cause biotic differentiation and biodiversity at multiple spatiotemporal scales. Recently, the collection of high‐resolution biological distribution data (e.g., specimens) and advances in analytical theory have led to the quantitative analysis and more refined spatial delineation of biogeographic regions. This study was conducted to redefine floristic zones in the southern part of the Korean Peninsula and to better understand the eco‐evolutionary significance of the spatial distribution patterns. Based on 309,333 distribution data of 2,954 vascular plant species in the Korean Peninsula, we derived floristic zones using self‐organizing maps. We compared the characteristics of the derived regions with those of historical floristic zones and ecologically important environmental factors (climate, geology, and geography). In the clustering analysis of the floristic assemblages, four distinct regions were identified, namely, the cold floristic zone (Zone I) in high‐altitude regions at the center of the Korean Peninsula, cool floristic zone (Zone II) in high‐altitude regions in the south of the Korean Peninsula, warm floristic zone (Zone III) in low‐altitude regions in the central and southern parts of the Korean Peninsula, and maritime warm floristic zone (Zone IV) including the volcanic islands Jejudo and Ulleungdo. Totally, 1,099 taxa were common to the four floristic zones. Zone IV showed the highest abundance of specific plants (those found in only one zone), with 404 taxa. Our study improves floristic zone definitions using high‐resolution regional biological distribution data. It will help better understand and re‐establish regional species diversity. In addition, our study provides key data for hotspot analysis required for the conservation of plant diversity.

## Graphical abstract

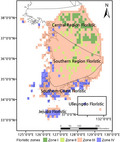

## 1 INTRODUCTION

The use of biota to investigate biogeographic regions, including their distribution patterns, is a key to better understand the ecological processes that create biotic differentiation and biodiversity at multiple spatiotemporal scales (Ricklefs, 2004). In particular, as an important step in understanding the spatial structure of biodiversity, delineating the distribution of these regions has been a foundation of basic and applied research in biogeography, ecology, earth science, and conservation ecology (Brum et al., 2017; Graham & Hijmans, 2006; Ibanez‐Erquiaga, Pacheco, Rivadeneira, & Tejada, 2018; Kreft & Jetz, 2010; Lenormand et al., 2019; Olson et al., 2001; Ricklefs, 2004; Sun, Yan, & Xie, 2008). Until the late 20th century, the proposed biological regions were based on limited data, convenience, and expert opinions (White, 1983). Without distinct criteria, the floristic zones were also suggested based on delineating endemic plant distribution (Takhtajan, 1986), accumulating floristic checklist data, and applying spatial statistics (McLaughlin, 1989). Recently, there have been several proposals for defining geographical spaces based on plants (Gonzalez‐Orozco et al., 2014; Kreft & Jetz, 2010; Lenormand et al., 2019; Vilhena & Antonelli, 2015). There are difficulties in precise mapping and understanding the regional patterns of biodiversity because of issues surrounding the reliability of floristic surveys (Daru et al., 2018; Geurin et al., 2018) and because flora is closely related to environmental gradients (landuse change and climate) and their complexity (Gonzalez‐Orozco et al., 2014). If these challenges can be overcome, the delineation of biological spaces based on reliable data will provide new metrics and perspectives through biogeographic regionalization (Lenormand et al., 2019).

The first attempt of biogeographic regionalization in the Korean Peninsula, made around 100 years ago, proposed convenient northern, central, and southern divisions based on the descriptions of plant and vegetation characteristics (Nakai, 1919). Subsequently, more finely differentiated floristic zones were defined by redistributing the divisions of Nakai (1919) (Lee & Yim, 1978). Pseudo‐floristic zones have been proposed from the perspective of vegetation and climate (Yim & Kira, 1975). All these floristic zones in the Korean Peninsula (including pseudo‐floristic zones), developed mostly from expert opinion or for convenience, were structured around homogeneous bands based on the relationship between latitude and mean annual air temperature. In the neighboring country China, large‐scale banded or planar vegetation‐climate zones have been delineated using a wetness index (Sun et al., 2008). However, regions with a complex mountainous structure show substantial changes in elevation and topography over short distances, and when this is combined with human influence, it results in complicated spatiotemporally driven biogeographic regions (see the map of floristic zones in the Korean Peninsula in Appendix S1) (Lenormand et al., 2019).

Given the lack of regionalization based on biological distribution data (e.g., specimens), pseudo‐biogeographical divisions have also been developed for conservation at large spatial scales (Olson et al., 2001; Sun et al., 2008). When delineating biogeographical zones, it is necessary to maximize the differences while also maximizing the homogeneity of the taxonomic assemblages among zones (Stoddart, 1992). Improving the analytical accuracy through the quantitative accumulation of organism distribution data, informatization of geography, and collection of large‐scale distribution data—which has been a challenge in the extraction of floristic and other biological zones—enable quantitative and rigorous regionalization (Linder et al., 2012). In recent studies, floristic zones have been delineated using accumulated data to incorporate information about plant distribution (Gonzalez‐Orozco et al., 2014). The reliability of point data for the distribution of organisms can be ensured only by using specimen data. Because of the use of global positioning systems (GPSs), plant specimens that provide spatial information can be collected. From the late 20th century, accurate and extensive plant lists and data on the distribution of specimens have been collected (e.g., Korea National Arboretum, 2016), and floristic regions are being defined at the regional and national levels using plant location data (Gonzalez‐Orozco et al., 2014; Korea National Arboretum, 2016; Lenormand et al., 2019). Therefore, approaches to delineate floristic zones using these data can be developed reliably and accurately. In particular, in the southern part of the Korean Peninsula, the plant specimens that have been collected and the distribution maps that have been composed since 2000 can be evaluated with reliable large‐scale data.

To analyze accumulated species distribution data, artificial neural networks (ANNs) are increasingly used as an alternative to traditional statistics to analyze multidimensional data (Chon, 2011; Cottrell, Olteanu, Rossi, & Villa‐Vialaneix, 2018; Snedden, 2019). Specifically, self‐organizing maps (SOMs), an ANN‐based technique using unsupervised learning, are suggested as an alternative to conventional primary component analysis (Ahn, Shin, Jeong, & Heo, 2018; Chon, 2011). Essentially, SOMs are classified as a non‐linear sequence analysis method, as the training data set is non‐linearly projected onto a less dimensional space (generally two dimensions) approximating the probability density function (Kohonen, 1995; Snedden, 2019). Unlike statistical approaches with mediator variables, SOMs do not make assumptions related to the correlations between variables and the distribution of variables (Chon, 2011; Giraudel & Lek, 2001; Snedden, 2019), and therefore, they are used with species presence or absence data (Céréghino, Santoul, Compin, & Mastrorillo, 2005; Paini, Worner, Cook, De Barro, & Thomas, 2010). Efforts to derive and visualize zones from plant species distribution data using conventional univariate statistical analyses have generally assumed the response of species data to environmental gradients, in accordance with Eigen‐based analytical approaches. However, these analyses are limited in cases when the shape of species abundance response (e.g., linear or unimodal) is not clear (Ahn et al., 2018; Liu, Kargupta, & Ryan, 2006; Snedden, 2019). Recently, numerous sets of distribution point data have been used for biological regionalization, but it is difficult to ascertain the relationships between species as variables. Self‐organizing maps reduce multidimensional data to two or three dimensions, making them useful for typification analysis using distribution points for a large number of plant species (including regionalization). Multidimensional scaling (MDS), which is typically used in the clustering analysis of existing species compositions with dimension‐reduction methods, together with background information and similarity (or dissimilarity) metrics, indicates characteristics that are sensitive to global dissimilarities (Kirt & Vainik, 2007). On the contrary, SOMs are more stressed by local similarities through flexible manifold approximation (Kirt, Vainik, & Võhandu, 2007). Furthermore, SOMs are suitable for extracting information from large datasets consisting of numerous sample units and variables at different scales unlike the conventional multivariate analysis (Park, Chon, Bae, Kim, & Lek, 2018). Although SOMs have limitations (Flexer, 1997), in this study, to clearly reflect local similarities among rare species (Warton, Wright, & Wang, 2012), we used SOMs with species presence–absence data.

Quaternary glacial–interglacial oscillations have been an important mechanism in shaping the current distribution of plants (Ricklefs, 1987). The Korean Peninsula, which is topographically composed of around 80% mountains, is characterized by a backbone mountain range (the Baekdudaegan Mountains) running from north to south with sub‐ranges branching off. The major mountains (≥1,000 m above sea level) are considered a single glacial refugium based on altitude rather than latitude, and they have a mixture of boreal and temperate flora (Chung, López‐Pujol, & Chung, 2017; Chung et al., 2018; Kim, Chung, Kim, Kim, & Lee, 2014). The botanical importance of peninsulas and mountainous regions is well established because of their topographic characteristics (Médail & Diadema, 2009). The mountains that form the core topography of the Korean Peninsula possess a floristic composition that is affected by latitude and spatiotemporal gradients, and this might be significant for its mutually distinct functions and evolutionary spaces. The flora of the Korean Peninsula is fundamentally controlled by a mixture of boreal and temperate abiotic conditions, and is affected, similar to other regions, by the agricultural and urbanization activities of humans.

The accumulation of a large volume of recent distribution point data and the application of analytical methods suited to the nature of the data have resolved the difficulties in floristic regionalization, enabling to propose rigorous floristic zones according to the actual distribution of species. However, it has been difficult to find case studies of accumulated, high‐resolution, georeferenced specimen data for plants, or to find studies related to geographic regionalization using an SOM. The present study was conducted to redefine the floristic zones in the southern part of the Korean Peninsula with SOMs and understand the eco‐evolutionary significance of the spatial distribution patterns. We used point distribution data for vascular plants collected at a high resolution in the southern part of the Korean Peninsula between 2003 and 2015. We aimed to (a) derive floristic delimitations, (b) identify the correlations with ecologically important environmental factors, and (c) discuss the eco‐evolutionary significance of the derived regions for floristic assemblages.

## 2 MATERIALS AND METHODS

### 2.1 Study region

**FIGURE 1:**
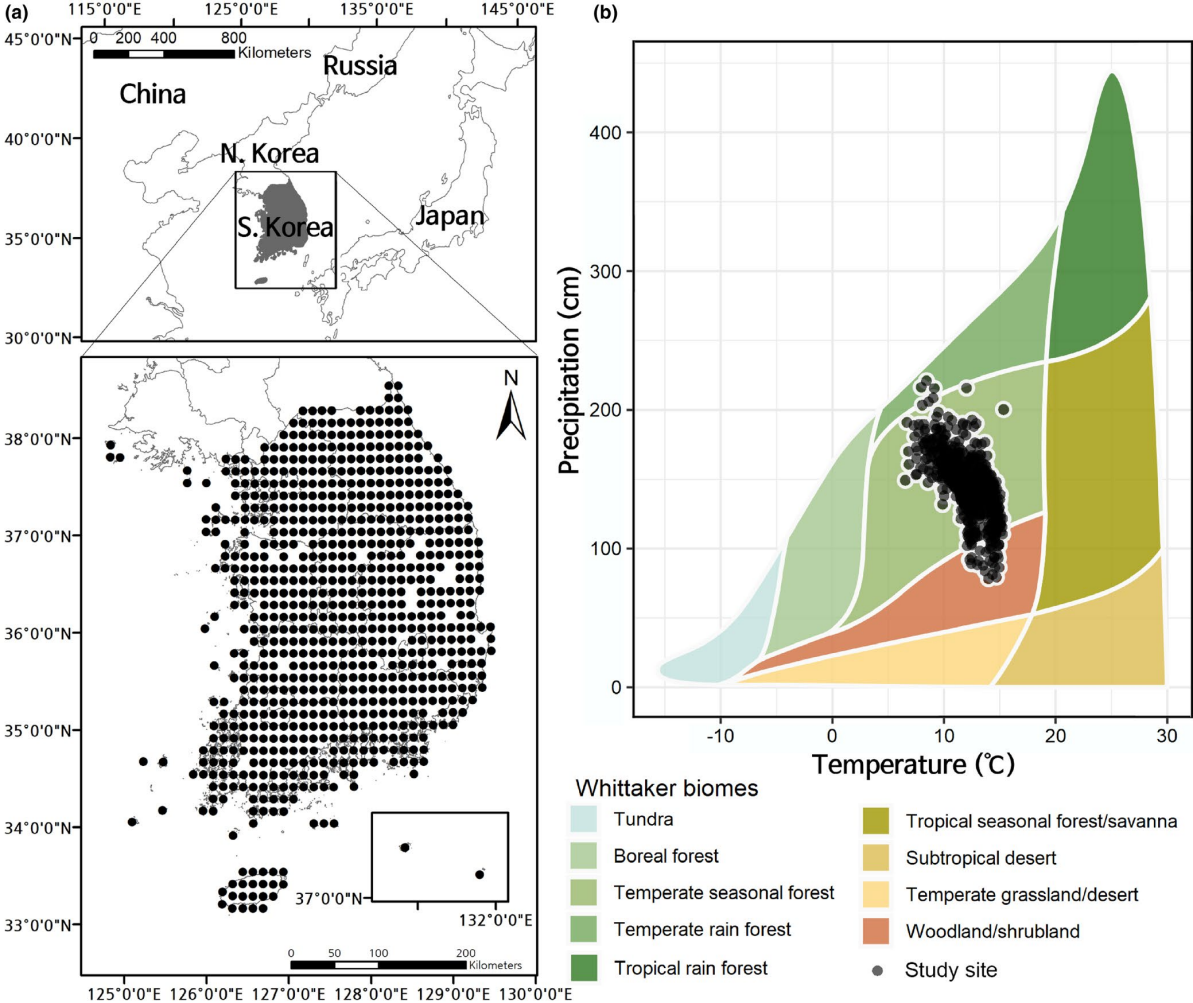
Representative study sites. (a) Distribution of study sites across the southern part of the Korean peninsula. (b) Distribution of study sites across the global vegetation biomes defined using the Whittaker classification

Our study was conducted in the southern part of the Korean Peninsula, which is located in the East Asia region (33°–38°N, 125°–131°E; Figure 1). The total area of the study region is 100,033 km^2^, and the human population is 51 million (Ministry of Land Infrastructure & Transport, 2016). The mean annual air temperature ranges from 10 to 15°C and the mean annual precipitation ranges from 1,000 to 1,900 mm (Korean Meteorological Administration, 2020).

The Korean Peninsula is situated adjacent to the west Pacific Ocean and is surrounded by water in three directions—east, west, and south. It is a temperate region with four distinct seasons associated with the East Asian monsoon that occurs in the far eastern side of the Asian continent (Yi, 2011). Winter (December‐February) is cold and dry because of the strong Siberian anticyclone from the Tibetan Plateau, whereas summer (June‐August) is hot and humid, with around 70% of the annual precipitation focused during this period (Korean Meteorological Administration, 2020) (Appendix S2). Although the Korean Peninsula is located at the eastern edge of the Eurasian continent, the humid air supplied from the Yellow Sea to the west affects the diversity and distribution of local plants.

The Korean Peninsula comprises numerous mountains centered in the Baekdudaegan Mountain Range, and only 22.5% of the peninsula is a flat land (Appendix S3) (Ministry of Land Infrastructure & Transport, 2016). In addition, although the elevation is not generally high, the region displays complex tectonic characteristics with a relatively diverse topography. Because the altitude gradients are shallower than those in other regions in East Asia, the borders between mountains and plateaus are relatively indistinct, making the region well suited for the spatiotemporal movement of plants (Ministry of Land Infrastructure & Transport, 2016).

There are approximately 4,300 known vascular plant species in the Korean Peninsula (with approximately 3,000 species in the southern part), including 280 pteridophyte species, 53 gymnosperm species, and 3,963 angiosperm species. *Pentactina*, *Echiosophora*, *Abeliophyllum*, *Hanabusaya*, *Mankyua*, and *Megaleranthis* are present as specialized genera. According to the Whittaker biome classification (Whittaker, 1962), the southern part of the Korean Peninsula is mostly occupied by temperate seasonal forest biomes, but it may also contain some temperate rain forest and woodland/shrubland biomes (Figure 1). There was a substantial loss of remnant forests, which were converted to grasslands and shrublands over approximately 600 years before the 20th century. Later, in the southern part of the Korean Peninsula, the South Korean Government implemented policies to promote forests from the 1970s, and consequently, most natural habitats are located in forests (Cho, Kim, & Koo, 2018). Currently, approximately 30.3% of the southern part of the Korean Peninsula is urbanized or used for agriculture and 63.8% is occupied by forests, with other land covers accounting for the remaining 5.9% (Ministry of Land Infrastructure & Transport, 2016).

### 2.2 Plant distribution data

We used vascular plant distribution data based on specimen and coordinate data of plants collected between 2003 and 2015 in the southern part of the Korean Peninsula (Korea National Arboretum, 2016). The vascular plant distribution maps contained coordinate data of 309,333 specimens (401 ± 21 specimens per sites), corresponding to 2,954 taxa in 175 families and 919 genera. For analysis, a grid system was overlaid on a national topographic map to combine the taxonomic groups located in each cell of the grid (cell size, 11.2 km × 13.9 km), with the location coordinates in a single data set (Graham & Hijmans, 2006; Lenormand et al., 2019). All 771 grid cells were used in the analysis, but some large urban regions were excluded from the floristic survey conducted by the Korea National Arboretum, and therefore, these were left as empty cells.

### 2.3 Analysis of floristic assemblage clusters and characteristics

**FIGURE 2:**
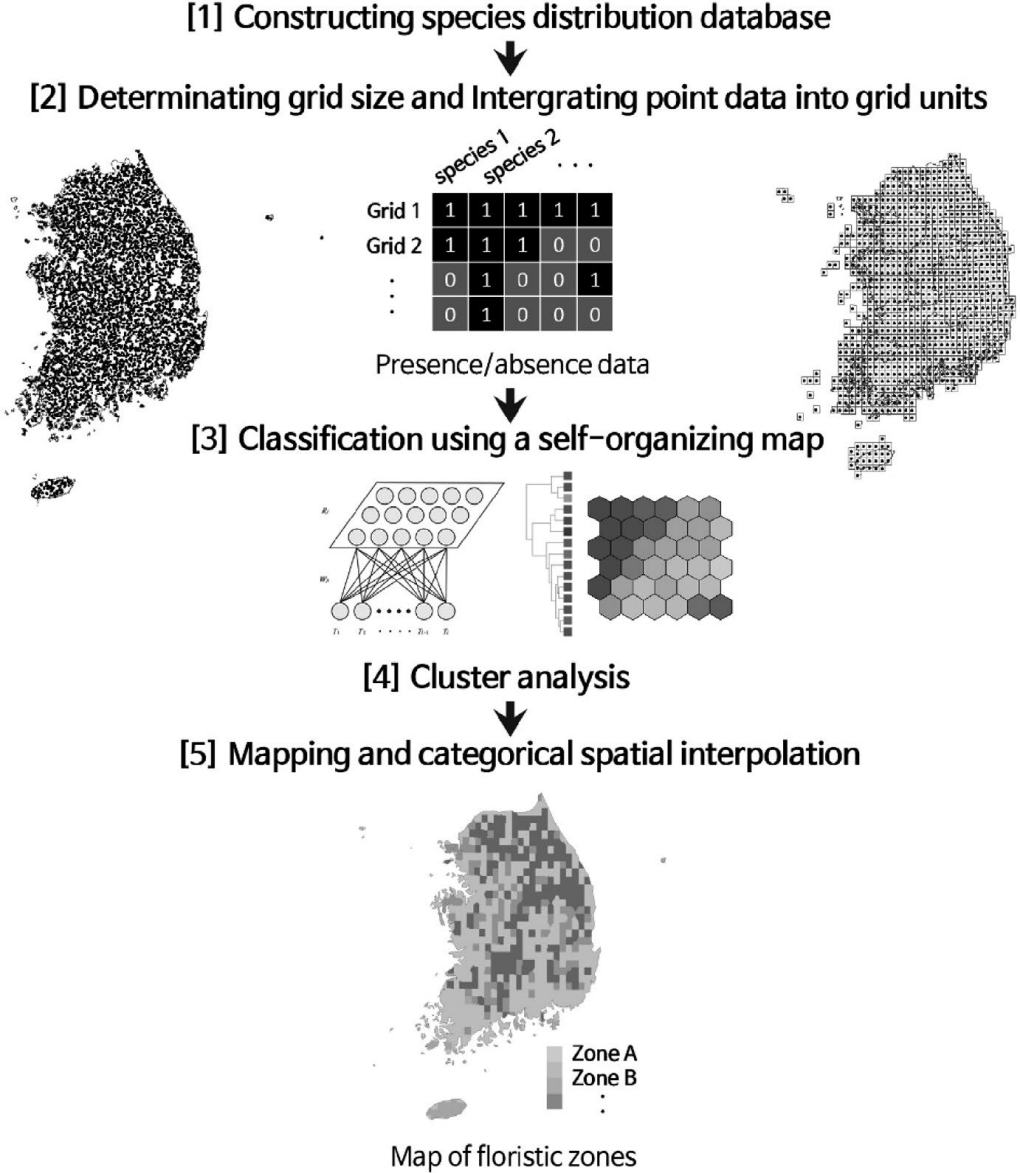
Conceptual diagram of the analysis steps and data flow in defining the floristic zones (Kreft & Jetz, 2010)

Using distribution data of the 771 grid cells and 2,954 plant taxa, a SOM training data set was constructed in the form of a presence–absence matrix (771 rows × 2,954 columns) (Figure 2). The kohonen R package was used for SOM algorithm (Wehrens & Kruisselbrink, 2018). We set a random seed because of random initialization of SOMs, and the output layer was composed of 81 output nodes arranged in a square lattice. To determine the types, the weight vectors of the SOM map units were subjected to a hierarchical cluster analysis after conversion to Euclidean distance metrics (via the function hclust in R using the complete linkage method). The optimal number of types was calculated by applying the silhouette coefficient in the range of 2–15 types (Rousseeuw, 1987). In mapping the regionalization results, the grid cells that were empty because of exclusion from the survey were filled using the maximum frequency value from the surrounding eight cells. As some island regions (Ulleungdo Island and Dokdo Island) showed heterogeneous values because of their distance from the adjacent grid cells, mapping was performed using type values within the local range.

The correlations in species composition among the floristic zones were analyzed using Venn diagrams (with the “VennDiagram” package) based on lists of species in each zone (Chen & Boutros, 2011). After listing all species in each zone, the common taxa (those appearing in all zones) and specific taxa (those appearing in only specific zones) were distinguished. Thereafter, floristic compositions were investigated by analyzing the identification of specific taxa at the family level.

### 2.4 Environmental data and analysis

Geographic and climatic factors were analyzed as macro‐environmental factors, using the defined floristic zones. For the geographic factors, the latitude and longitude were used, and for climatic factors, air temperature and precipitation data—provided by the Korean Meteorological Administration (2020) and collected from 583 points between 1970 and 2010—were used. In addition to the direct environmental data, the warmth index (WI) and coldness index (CI) were calculated and used as indirect climate data (Kira, 1945) (Equations 1 and 2). The values for these environmental factors were converted to values covering the entire southern part of the Korean Peninsula by linear interpolation, accounting for topography and altitude, using ArcGIS program (ver. 10.0). The mean value of the environmental factors in each grid cell was then calculated and used in the analysis.

(1) $$\text{WI}=\sum_{1}^{n}\left(t‐5\right):t>5$$

(2) $$\text{CI}\;=\;‐\sum_{1}^{n}\left(t‐5\right):t<5$$

Equations 1 and 2. *t*: mean monthly temperature (°C).

The physical factors affecting plant distribution, parent materials, topography, effective soil depth, and soil texture in the southern part of the Korean Peninsula were used (Rural Development Administration, 2010). Parent materials were categorized as acidic rock, metamorphic rock, sedimentary rock, quaternary deposit, volcanic ash, and other. Topography was categorized into mountain, hill, pediment, interrill area, fan, lava terrace, or other. Effective soil depth was categorized into four classes (<20, 20–50, 50–100, and >100 cm). Soil texture was categorized as sandy gravel, silt and sandy loam, clay loam, and clay (Appendix S4).

To test the effect of environmental factors on the floristic composition and zonation, the geographic and climatic (mean annual temperature, annual precipitation, warmth index, and coldness index) data were analyzed using the one‐way analysis of variance (ANOVA) and Tukey's test (Zar, 1984). The categorical physical factors (parent materials, topography, effective soil depth, and soil texture) were analyzed using box plots for each zone. The “ggplot2” R package was used for data visualization (Wickham, 2016). Statistical analyses were performed using R (R Core Team, 2019).

## 3 RESULTS

### 3.1 Flora extraction and taxon distribution

**FIGURE 3:**
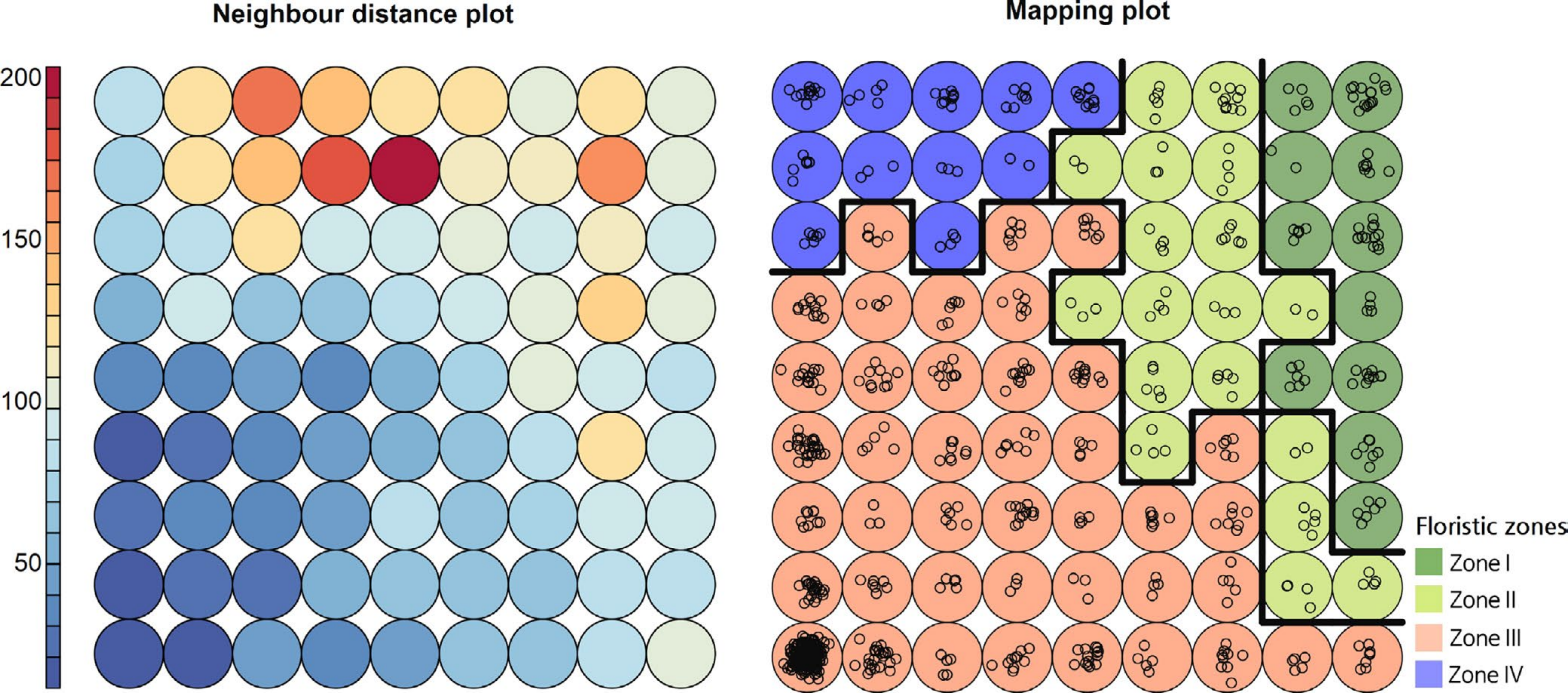
Results of the self‐organizing map (SOM) analysis for 9 × 9 SOM map. (a) The neighbor distance plot or U‐Matrix indicating the distance between each node and its neighbors. (b) The classification of the training samples according to SOMs

**FIGURE 4:**
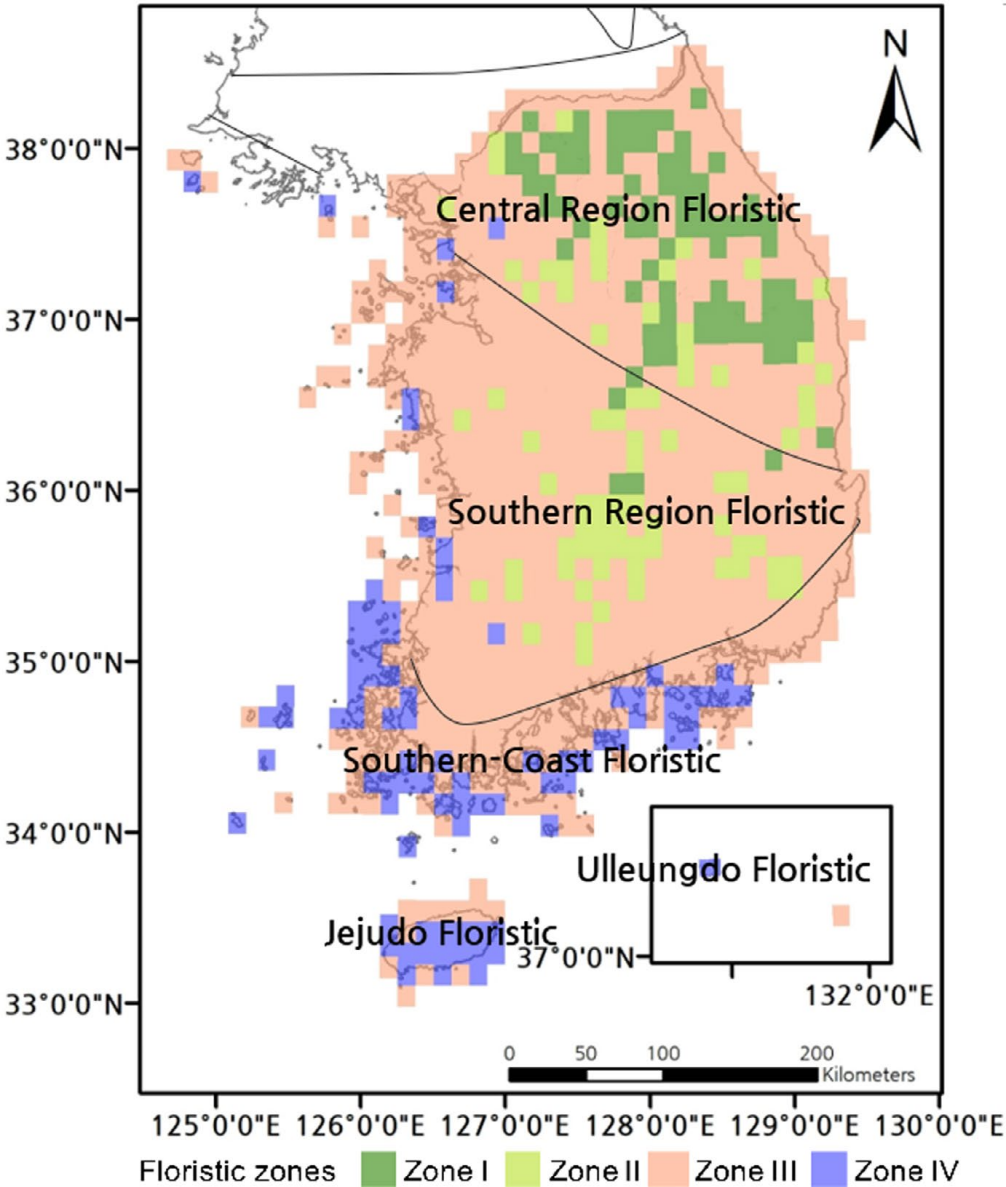
Four floristic zones mapped to the southern Korean Peninsula, derived using plant distribution data. The straight and solid lines represent the named historical floristic regions. The lines dividing the historical floristic regions have previously been used to explain the observed distribution of plants in the Korean Peninsula (e.g., north, central, and southern Korean Peninsula) (Lee & Yim, 2002)

After the conversion of coordinates of the plant specimens to floristic composition data for the 771 grid cells, the phytogeographic structure of the southern part of the Korean Peninsula was divided into four zones (maximum silhouette coefficient, 0.7968; Table 1, Figures 3 and 4). The peninsula was divided into three inland zones—the cold floristic zone (hereinafter, Zone I), corresponding to the high‐altitude regions in the central part of the peninsula; the cool floristic zone (hereinafter, Zone II), corresponding to the high‐altitude regions in the southern part of the peninsula; the warm floristic zone (hereinafter, Zone III), corresponding to the lowlands in the central and southern parts of the peninsula; and the maritime warm floristic zone (hereinafter, Zone IV), including Jejudo Island and Ulleungdo Island.

**TABLE 1:** Geographical and biological statistics used to delineate the four floristic zones in the southern Korean Peninsula

Floristic zone	Area (km^2^) (%)	Species richness	Mean species richness (per grid) (± *SE*)
Zone I (Cold floristic zone in central high elevation areas)	12,419 (12.3)	1,700	464.7 ± 9.9
Zone II (Cool floristic zone in central and southern high elevation areas)	11,619 (11.5)	1,668	331.7 ± 11.5
Zone III (Warm floristic zone in central and southern hilly areas)	72,367 (72.0)	2,379	79.3 ± 3.4
Zone IV (Maritime floristic zone near the coast and islands)	4,265 (4.2)	2,200	298.6 ± 14.0
Total	100,378 (100.0)	2,954	162.6 ± 5.8

Although these zones formed large patches and occupied large areas, smaller patches were scattered within the other floristic zones (Figure 4). This is because Zone III, which is connected to Zone I, contains most of the major cities in a given region or broadscale production (forestry and agriculture) regions. Our study region also included the military demarcation line between North and South Korea. The region near the military demarcation line (near the 38th parallel, length, 248 km; area, 907 km^2^) is characterized by a high level of disruption and environmental management activities.

Zone III—broadly covering the lowlands of the southern part of the Korean Peninsula—occupied the largest area (72.0%), followed by Zones I (12.3%) and II (11.5%) in regions with high‐altitude mountains, and Zone IV (4.2%), which includes coastal and island regions (Table 1, Figure 4). Unlike the area gradient, the order of species abundance (from the highest to lowest) was as follows: Zone III (2,379 taxa), Zone IV (2,200 taxa), Zone I (1,700 taxa), and Zone II (1,668 taxa). The species abundance per classified grid cell was the highest in Zone I (464.7 ± 9.9 taxa), followed by Zone II (331.7 ± 11.5 taxa), Zone IV (298.6 ± 14.0 taxa), and finally Zone III (79.3 ± 3.4 taxa), which contains a large number of developed regions.

### 3.2 Geographic range and climatic environment in each floristic zone

**FIGURE 5:**
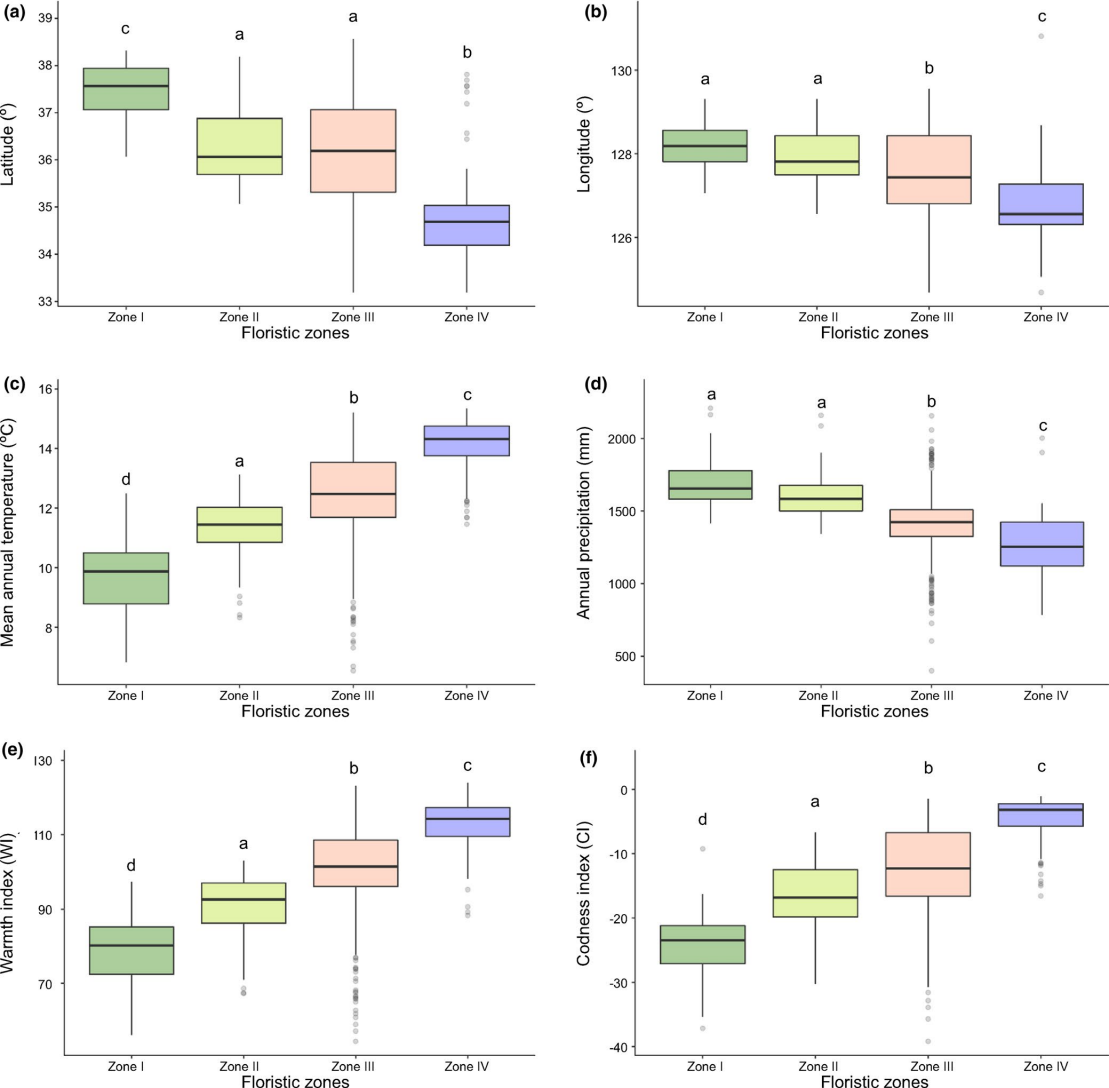
Comparison of geographic and climatic factors in the derived floristic zones in the southern Korean Peninsula. (a) Latitude, (b) longitude, (c) mean annual temperature, (d) annual precipitation, (e) warmth index, and (f) coldness index. Median values are marked inside the boxplots; the errors bars indicate the 90th and 10th percentiles and the points outside of the error bars indicate the outliers. A Tukey post hoc test revealed significance groups, represented by letters (*p* < .05)

All the floristic zones showed significant differences in the mean longitude and latitude, mean annual temperature, annual precipitation, WI, and CI per grid cell (*p* < .001, Figure 5a–f). While Zone I is focused in the central part of the Korean Peninsula, Zone II is mostly situated in the center of the southern part of the peninsula, although there are scattered small patches within the central region adjacent to Zone I or inside Zone III (Figures 4 and 5a–b). Zone IV is mostly located on the coast and islands in the west and south of the peninsula, as well as some inland areas in the west. Zone III showed a relatively broad longitudinal and latitudinal range, as it was distributed throughout the study region across the entire southern part of the Korean Peninsula.

When the climatic environments were compared between the floristic zones, differences were observed in the mean annual air temperature, mean precipitation, WI, and CI (Figure 5c–f). The mean annual air temperature showed a gradually increasing trend from Zones I to IV. The annual precipitation showed the opposite trend, but there was no clear difference between Zones I and II. As indirect climate factors, the mean WI and CI also showed clear differences among the zones. In accordance with its wide geographic distribution, Zone III showed the widest ranges for these climate factors.

### 3.3 Physical environment in each floristic zone

The parent materials in the southern part of the Korean Peninsula are predominantly acidic rocks (48.5%) and metamorphic rocks (26.3%), and the ratio of plateaus is very low. The most common soil depth is 50–100 cm (39.1%), and 93.7% of the soil consists of silt and sandy loam (51.6%) or clay loam (42.1%) (Ministry of Land Infrastructure & Transport, 2016) (Appendix S4).

**FIGURE 6:**
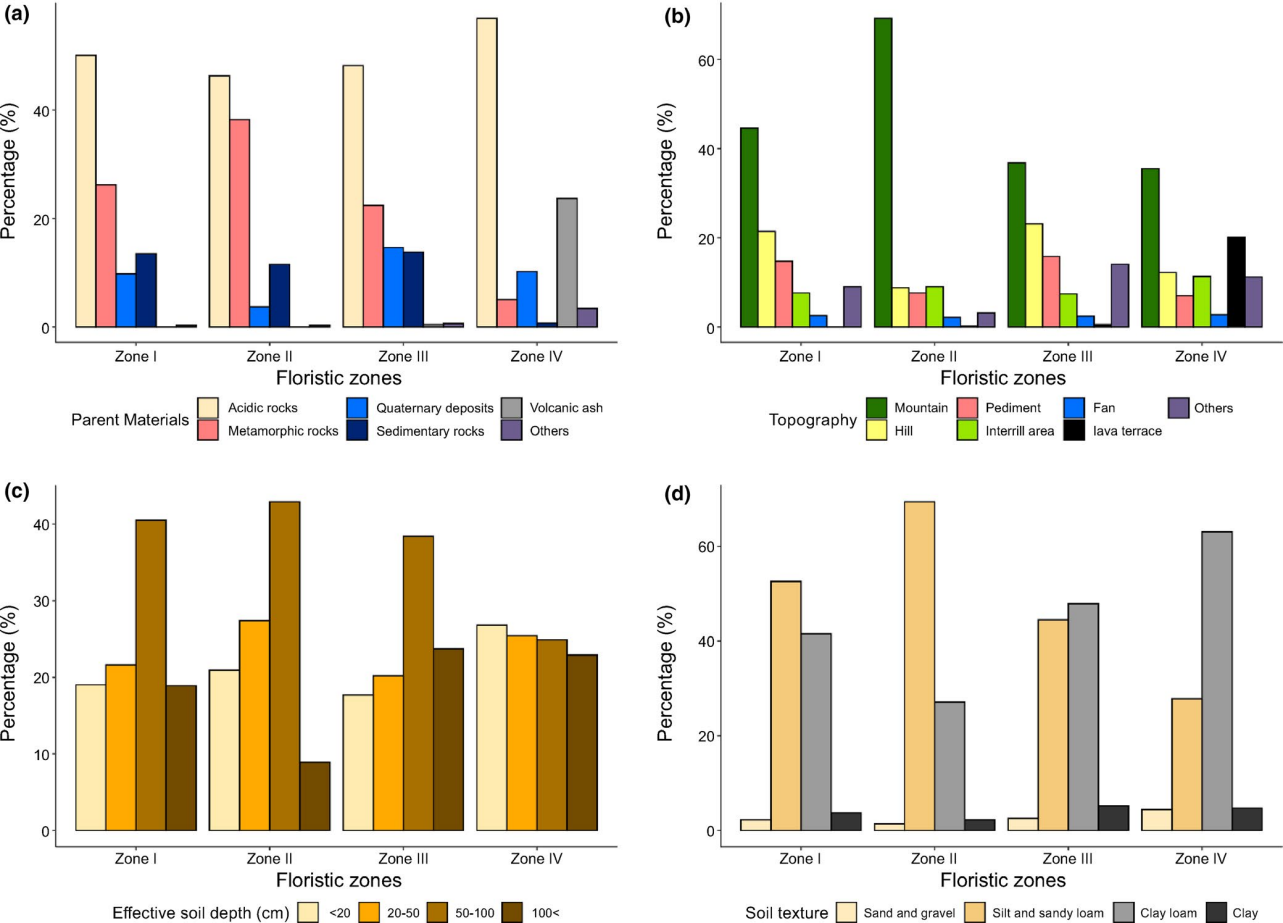
Analysis of composition ratios of physical factors in each floristic zone in the Korean Peninsula. (a) Parent material, (b) topography, (c) effective soil depth, and (d) soil texture

The parent materials across the four floristic zones consisted of over 70% acidic and metamorphic rocks; however, Zone IV, which includes the volcanic island Jejudo and the oceanic island Ulleungdo, showed a high ratio of volcanic ash (23.7%) among its parent materials (Table 2). In addition, the terrain type and soil type in Zone IV showed a considerably higher proportion of lava terrace and clay loam, respectively, and the soil depth classes were more evenly distributed compared with the other zones (Figure 6).

**TABLE 2:** Analysis of the composition ratio (%) of physical environmental factors in the research area and derived floristic assemblage zones, including topography, soil parent materials, soil depth, and soil type

Parameter	Zone I	Zone II	Zone III	Zone IV	Total land area
Topological class					
Mountain	44.6	69.2	36.8	35.5	46.9
Hill	21.4	8.8	23.1	12.2	18.2
Pediment	14.7	7.6	15.8	7.0	12.8
Interrill area	7.6	9.0	7.4	11.3	8.1
Fan	2.5	2.1	2.4	2.7	2.3
Lava terrace	–	0.2	0.5	20.1	1.5
Others	9.0	3.1	14.0	11.2	10.1
Sum	100.0	100.0	100.0	100.0	100.0
Parent material type					
Acidic rocks	50.1	46.3	48.2	56.9	48.5
Metamorphic rocks	26.2	38.2	22.4	5.0	26.3
Sedimentary rocks	13.5	11.5	13.8	0.7	12.3
Quaternary deposits	9.8	3.7	14.6	10.2	10.6
Volcanic ash	–	–	0.4	23.7	1.7
Others	0.3	0.3	0.6	3.4	0.6
Sum	100.0	100.0	100.0	100.0	100.0
Soil depth class (cm)					
<20	19.0	20.9	17.7	26.8	19.3
20–50	21.6	27.4	20.2	25.4	22.7
50–100	40.5	42.9	38.4	24.9	39.1
>100	18.9	8.9	23.7	22.9	18.8
Sum	100.0	100.0	100.0	100.0	100.0
Soil texture type					
Sandy gravel	2.2	1.4	2.5	4.4	2.3
Silt sandy loam	52.6	69.4	44.5	27.8	51.6
Clay loam	41.5	27.1	47.9	63.1	42.1
Clay	3.7	2.2	5.2	4.7	4.1
Sum	100.0	100.0	100.0	100.0	100.0

### 3.4 Relationships between species composition in each floristic zone

**FIGURE 7:**
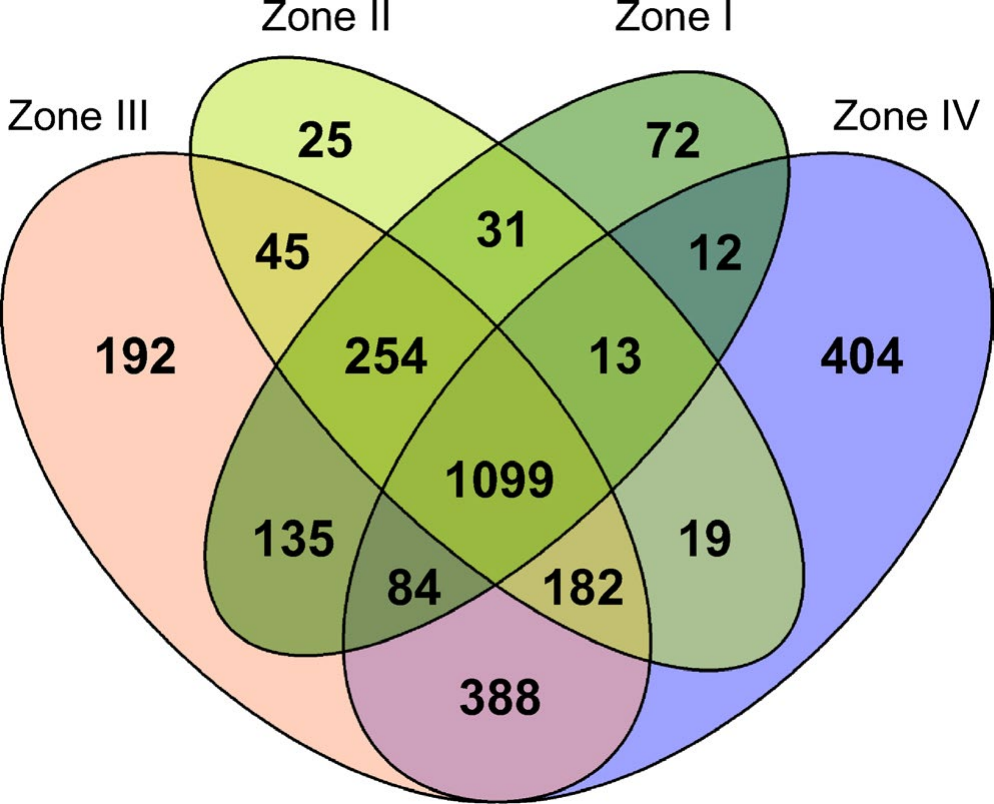
Venn diagram showing the relationships between plant distributions in each of the four floristic zones. Numbers represent individual taxonomic groups

There were 1,099 common taxa in all zones (Figure 7). Interestingly, the zone with the most specific taxa was Zone IV (404 specific taxa, 18.4%), which had the smallest area. This was followed by Zone III (192, 8.1%), Zone I (72, 4.2%), and Zone II (25, 1.5%).

**FIGURE 8:**
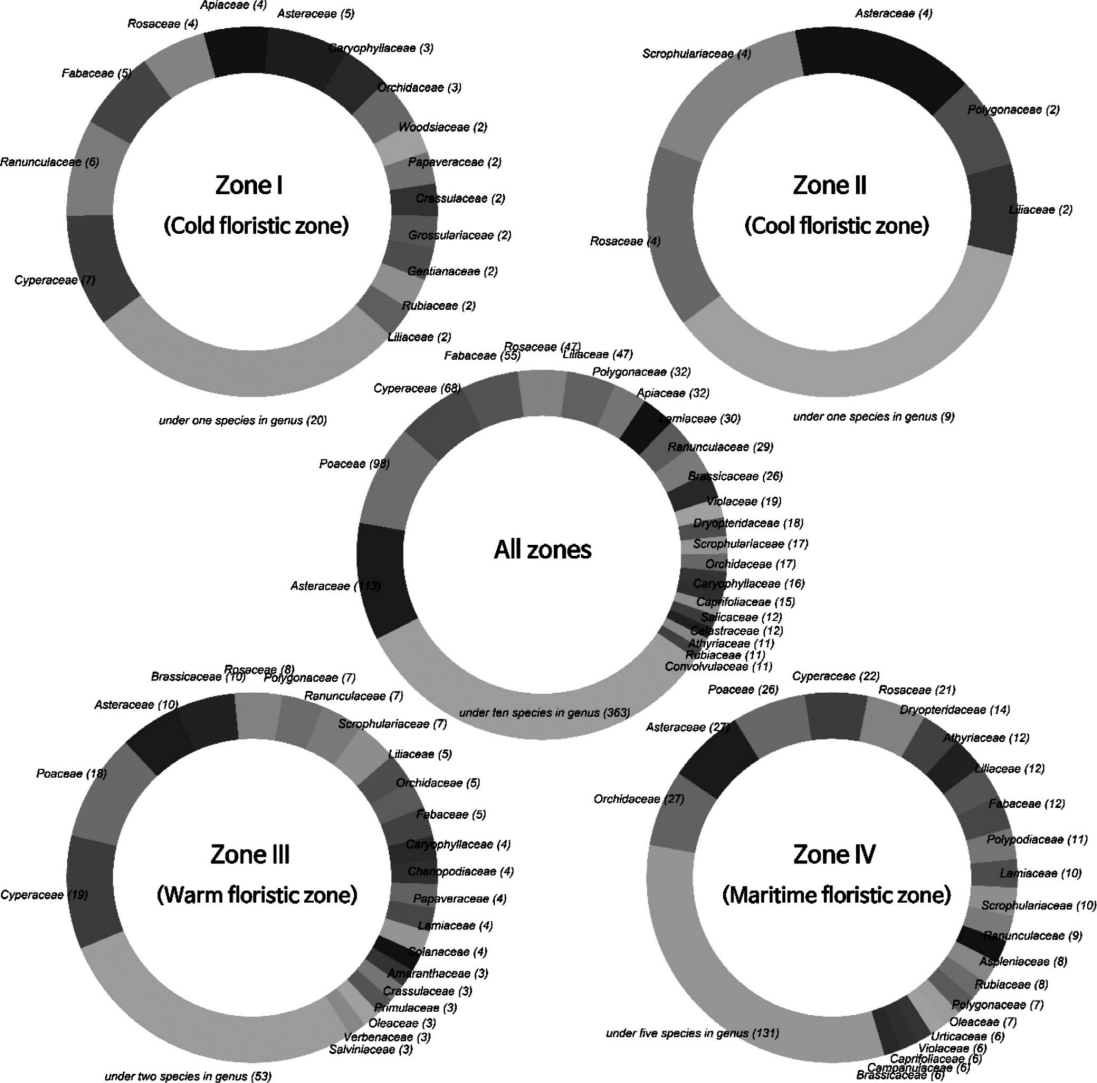
Comparison of the composition of specific plant families in each floristic zone. As shown in Figure 7, there were 1,099 taxa that appeared in all four zones. The specific plant species in Zones I, II, III, and IV were 72, 25, 192, and 404 taxa, respectively

When specific taxa were analyzed at the family level (Figure 8), Zone III—generally consisting of low‐lying hilly terrain heavily affected by human activity and development—showed a higher diversity of Poaceae (18 out of 192 taxa) than Zones I and II, whereas marine and coastal Zone IV were characterized by a high diversity of Orchidaceae (27 out of 404 taxa), Asteraceae, and Rosaceae. Among the common species appearing in all zones, the families with the highest diversity, in descending order, were Asteraceae (113 taxa), Poaceae (98 taxa), Cyperaceae (68 taxa), Fabaceae (55 taxa), and Rosaceae (47 taxa) (Figure 8).

## 4 DISCUSSION

Historically, biogeographic spaces have predominantly been delineated using the distribution of organisms according to specific knowledge (e.g., endemism and evergreen trees), or the corresponding geographical and climatic factors, rather than using the actual recorded biota (Takhtajan, 1986; White, 1983). Historical definitions of vegetation climate and floristic zones in the Korean Peninsula have used these methods (Lee & Yim, 1978, 2002; Yim & Kira, 1975). Recently, there have been attempts to review bioregions using quantitative analytical techniques, with some showing similar and some different results to the historical regions (Gonzalez‐Orozco et al., 2014; Lenormand et al., 2019). The present study fundamentally differs from previous approaches to phytogeographic regionalization because we used high‐resolution, georeferenced specimen data for the southern part of the Korean Peninsula. Although restricted to the southern regions, our results provide data‐based phytogeographic zones using SOMs and georeferenced data for recently collected plant specimens. The spatial distribution of floristic zones and the basic taxonomic composition in the southern part of the Korean Peninsula reflects a combination of repeated migrations, expansions, and reductions in species associated with climatic variation, physical and geographic factors, and anthropogenic effects (Valladares, Bastias, Godoy, Granda, & Escudero, 2015). In addition, we reconfirmed differences identified in historical studies in geographic and climatic conditions, which are determining factors of the spatial patterns of floristic assemblages (Lee & Yim, 1978, 2002; Yim & Kira, 1975). We also verified the correlations between zones and zone distribution based on the characteristics of the floristic assemblages and the physical conditions where they are found, such as the topographic characteristics of the Korean Peninsula. In particular, we verified the importance of the topography of the Korean Peninsula (especially the connected mountainous regions and isolated mountains), which has been emphasized in recent studies (Chung, López‐Pujol, et al., 2017), and its contribution to shifts in distribution related to climate change and the process of floristic reassembly. Furthermore, the deterministic factors of floristic assembly used historically (e.g., climatic factors) and more diverse ecologically important environmental factors should be considered at the same distribution pattern as the southern part of the Korean Peninsula.

### 4.1 Characteristics of the new floristic zones

The changes in the flora of the southern Korean Peninsula (from warm to cool) and the effects of physical and human activities were investigated via the derived floristic zones. The historical floristic zones of the Korean Peninsula were constructed to broadly reflect a combination of climatic factors and plant distribution patterns, and showed a banded, near‐planar shape (Appendix S1) (Lee & Yim, 1978). Our SOM results, revealing four statistically significant spatial clusters (Zones I–IV) representing distinct territories (detailed below), were consistent with unification (or partial inclusion) or division of the historical zones. The historical central and southern zones partially reflected the distribution patterns of some characteristic trees, including evergreen broad‐leaved trees (e.g., *Citrus* and *Ilex*), but there was limited consideration of important biogeographic factors in small regions, such as complex mountain groups and the effects of altitude. Rather than forming a broad banded pattern, recent studies have revealed spatial patterns (at the global and regional scales) that include a large number of small, heterogeneous patches within larger biotic assemblage zones, arising from changes in the climatic and physical environment, such as quaternary glacial–interglacial oscillations, and the roles of factors such as topography (Kreft & Jetz, 2010; Lenormand et al., 2019; Médail & Diadema, 2009; Silva & Souza, 2018).

Zone I is focused around high‐altitude mountains in the central Korean Peninsula (maximum altitude: 1,708 m, Mt. Seoraksan) and included some high‐altitude regions in the south. Among the historical zones, this partially corresponds to the central and southern zones. Zone I reflects floristic elements that moved south from the previous periglacial environment and remained in the high‐altitude mountain ranges in the center of the Korean Peninsula after the recession of the periglacial environment. In East Asia, this region represents the southern limit of the ranges of the dwarf Siberian pine (*Pinus pumila*), Korean arborvitae (*Thuja koraiensis*), and Khingan fir (*Abies nephrolepis*) (Kong, Kim, Kim, Lee, & Shin, 2019; Korea National Arboretum, 2015). It is also an area with active cool‐climate highland agriculture and timber production (*P. koraiensis* and *Larix kaempferi*).

Zone II is focused on mountainous land adjacent to Zone I, mostly consisting of inland, high‐altitude mountains further south in the Korean Peninsula (1,915 m, Mt. Jirisan and 1,614 m, Mt. Deogyusan). Among the historical zones, this zone partially corresponds to the same central and southern floristic zones as Zone I. This region is the northern limit of the range of the Korean fir (*Abies koreana*) and the southern limit in East Asia of the range of dark‐bark spruce (*Picea jezoensis*) (Korea National Arboretum, 2015). Through the same historical geographical processes as in Zone I, this zone shows remnants of influence of the periglacial environment in the southern Korean Peninsula. In addition, trees that are mostly distributed in warmer or maritime climates (e.g., *Stewartia koreana* and *Lindera sericea*) can be found growing in the medium‐ and high‐altitude areas of Zone II (Kim et al., 2014). There is almost no highland agriculture in this zone, but some timber production occurs (mostly *Larix kaempferi*), and the zone is adjacent to numerous large cities.

Zone III accounts for most of the southern Korean Peninsula. Although this zone contains tall mountains and mountain ranges, it is also subject to complex effects from agricultural activity and cities with a relatively intensive level of land use. For example, the inclusion of areas in Zone III with higher latitudes than Zone I is likely because of the effect of long‐term military activity in these areas. Notably, in Zone III, the plants constituting the other floristic zones (Zones I, II, and IV) remain in isolated islands. These heterogeneous patches within Zone III are a vestige of shifts in plant diversity patterns driven by historical geographical changes, and thus, they are important for the heterogeneous formation of the regional floristic composition (Laliberté, Zemunik, & Turner, 2014; Zobel, 1997). Recently, the function of the Korean Peninsula as a shelter for biodiversity has been emphasized, because of its topography that includes several core mountains (Chung et al., 2018). In‐depth studies should be conducted on the biodiversity conservation functions (e.g., provision of shelter) of these small, heterogeneous patches and on their long‐term changes.

Zone IV unifies the historical floristic zones of the southern coast, Jejudo Island, and Ulleungdo Island, which have previously been more finely divided (Appendix S1), and has a high relative abundance of Orchidaceae, Asteraceae, and Rosaceae as well as specific taxa that only appear in this zone. Although Zone IV includes some inland areas, it mostly consists of regions along the coast of the Korean Peninsula and islands that are important for biodiversity, such as the oceanic island Ulleungdo (Chang & Gil, 2014; Choi, Yang, Yang, & Friesen, 2019; Holman et al., 2017) and the volcanic island Jejudo, which contains Hallasan Mountain (1,950 m). This zone ranges from the temperate zone of evergreen broad‐leaf trees (e.g., *Castanopsis sieboldii*) to the cool zone of polar trees (e.g., *Diapensia lapponica* var. *obovate*). Importantly, among the four identified floristic zones, Zone IV shows a relatively high diversity of specific taxa, and is a core part of the range of Orchidaceae, making it an important zone from a conservation and evolutionary perspective. Because this zone includes the volcanic islands Ulleungdo and Jejudo, the physical conditions considerably differ from those of the other zones, including parent materials (volcanic ash) and terrain (lava terraces). The north of Jejudo has acted as an agricultural and administrative center for longer than the south, and this explains the differences in the flora.

### 4.2 Spatial clustering and separation of floristic assemblages

The Korean Peninsula is composed of a network of mountain ranges along a latitudinal gradient and has witnessed interactions among the Manchurian flora region at higher latitudes, the North Chinese flora region at lower latitudes, and the Japan‐Korean flora region (Takhtajan, 1986). This is the background for the current plant diversity and species composition in the peninsula (Appendix S2). Thus, through repeated historical geographical processes, such as periglacial climates, the Korean Peninsula has acted as a geographical and biological corridor, with a mixture of high‐ and low‐latitude plants; this has resulted in the present‐day spatial distribution of biodiversity (Chang, Kim, Son, & Kim, 2016; Chung, Chang, et al., 2017; Chung, López‐Pujol, et al., 2017; Kim et al., 2014; Kim, Hwang, Lee, Yang, & Gorovoy, 2005; Kong et al., 2019).

Our revised floristic zones in the southern part of the Korean Peninsula reveal a new pattern, with Zones I and II forming central regions surrounded by a background of Zone III, with small patches of Zones I and II within Zone III. At the regional scale (e.g., the Korean Peninsula), complex physical and topographic factors can affect biotic assemblages (Lasmar et al., 2020; Tsiftsis, Tsiripidis, Karagiannakidou, & Alifragis, 2008; Xu, Zhang, Tian, Zeng, & Huang, 2016). Across a broader area, there are also effects of climatic factors, such as latitude (Sanders, Lessard, Fitzpatrick, & Dunn, 2007). The sequential settlement, expansion, contraction, and maintenance of species with historical geographic environmental oscillations are major processes involving interactions between the biota and topographic locations in a given region; this has been reported in other ecological regions (Lenormand et al., 2019; Silva & Souza, 2018). The national parks in Mt. Mudeungsan (1,187 m) and Mt. Gyeryongsan (846 m) in the center of the southern Korean Peninsula, the high‐altitude Mt. Palgonsan (1,192 m) region in the southeast Korean Peninsula, and the Youngnam Mountains (an aggregation of nine mountains over 1,000 m) are important locations containing local‐scale plant communities (e.g., *A. koreana*, *Primula farinosa*, and *Carex tenuiformis* in the Youngnam mountains), which are distant from the heterogeneous flora and the central distribution of Zone III (Kim et al., 2015; Korea National Arboretum, 2014). The mountains in these regions are an important factor in the formation of heterogeneous, small‐scale biological interactions (Gentili et al., 2015; Thomson, 2005) and the process of species differentiation.

Excluding the oceanic island Ulleungdo, the islands in the Korean Peninsula were last connected to the mainland before the Early Holocene, around 7,000 years ago (Kim, Kim, Won, Kim, & Kong, 2016). The islands have spent a long time, in terms of evolutionary biology, separated from the East Asian mainland. Among the zones defined in this study, the coastal regions and islands that constitute Zone IV (the warmest zone but with the least annual precipitation) showed that the abundance of certain plant species was high (but low overall mean abundance) and specific plants (e.g., Orchidaceae and Asteraceae) were diverse. As discussed above, these results were likely because of the inclusion of the volcanic island Jejudo (1,950 m above sea level) with its uniquely warm and humid maritime climate, and the oceanic island Ulleungdo (986.7 m above sea level). Orchidaceae (71.4%, e.g., *Habenaria chejuensis*) and Asteraceae (24.3%, e.g., *Artemisia hallaisanensis*) that are only present in Zone IV include species that only grow on Jejudo Island or Ulleungdo Island. Orchidaceae are the most abundant in warm and humid regions and show a negative correlation with the latitudinal gradient (Cribb, Kell, Dixon, & Barrett, 2003; Myers, Mittermeier, Mittermeier, da Fonseca, & Kent, 2000). This family can be a biodiversity indicator, as the members show specialized habitat preference (Cho, Kim, Koo, & Shin, 2019) and form associations among multiple species, including pollinators and mycorrhizal fungi (Pemberton, 2010). Spatial separation (as observed on isolated islands) is a core mechanism of species differentiation, but concomitant ecologically important environmental variables (e.g., climatic and physical conditions) also operate in combination with spatial separation to create selection pressures (Anacker & Strauss, 2014). Despite its narrow width, Zone IV is a key region in terms of ecologically important environmental characteristics, taxon diversity, and species differentiation, and thus, among the floristic zones in the Korean Peninsula, it occupies an important position from a conservation and evolutionary perspective.

Away from the coast, the mainland regions in Zone IV include Mt. Bukhansan, which is a national park close to the western coast, and Mt. Mudeungsan, which is in the southwest mainland. These regions are large mountains within Zone III and are thought to be vestiges after the entry of coastal and island floristic features from a past environment. The slopes of Mt. Mudeungsan have many warm wind holes (Park, 2017) and contain numerous plants that can otherwise be found mostly along the coasts and in islands (e.g., *Cyrtosia septentrionalis* (Rchb. f.) Garay and Polypodiales) (Hong et al., 2013). Some islands to the south and west of the Korean Peninsula were categorized into Zone III. A landscape that includes grazing and crop farming predominates the islands in the south of the Korean Peninsula, and thus, there has been an active introduction of plant species from the mainland, changing the flora considerably over time (Kim, Kim, Jeon, Kim, & Kong, 2017). These ecological landscape characteristics could form a background for the high floristic connectivity of some islands within Zone III, which mostly has flora in inland developed regions and warm temperate climates.

There are some limits in explaining the processes and significance of spatial clustering and separation of floristic assemblages using regional patterns alone. Detailed descriptions of the plant reassembly process after the last glacial maximum and epoch‐scale studies, such as pollen analysis, are needed (Yi, 2011). In addition, in the derived floristic zones, a convergent approach to phylogenetic history and diversity would be particularly useful for analyzing historical incidents in the formation of current biodiversity patterns and for ascertaining the historical and evolutionary relationships between zones. Nevertheless, deducing the spatial arrangements of floristic assemblages is of great importance for understanding the ecologically important environmental factors involved in forming biogeographic regions. Our study provides essential background information to develop precise conservation strategies based on micro‐ (Fenu, Mattana, Congiu, & Bacchetta, 2010) and nano‐hotspots (Grant & Samways, 2011) at the local scale. Moreover, there is great potential to quantitatively calculate the rarity, endemicity, and commonness of plant species, and to improve the priority of conservation and research (Casazza, Barberis, & Minuto, 2005).

## CONFLICT OF INTERESTS

The authors declare that they have no competing interests.

## AUTHOR CONTRIBUTION

**Songhie Jung:** Formal analysis (lead); Methodology (equal); Writing‐original draft (lead). **Yong‐chan Cho:** Conceptualization (lead); Methodology (equal); Writing‐review & editing (lead).

### OPEN RESEARCH BADGES

This article has been awarded Open Materials, Open Data, Preregistered Research Badges. All materials and data are publicly accessible via the Open Science Framework at https://doi.org/10.5061/dryad.3tx95x6cr.

## Supporting information

AppendixS1–S4

## Data Availability

The datasets generated and/or analyzed in the present study are available in Dryad at https://doi.org/10.5061/dryad.3tx95x6cr.
