# Supplementary material for: Redefining floristic zones in the Korean Peninsula using high‐resolution georeferenced specimen data and self‐organizing maps
Source: Ecol Evol. 2020 Sep 24;10(20):11549–64. doi: 10.1002/ece3.6790 (PMC7593177; doi:10.1002/ece3.6790)
Supplement: Supplementary file 1 — AppendixS1–S4 [file ECE3-10-11549-s001.docx]

**Appendices**


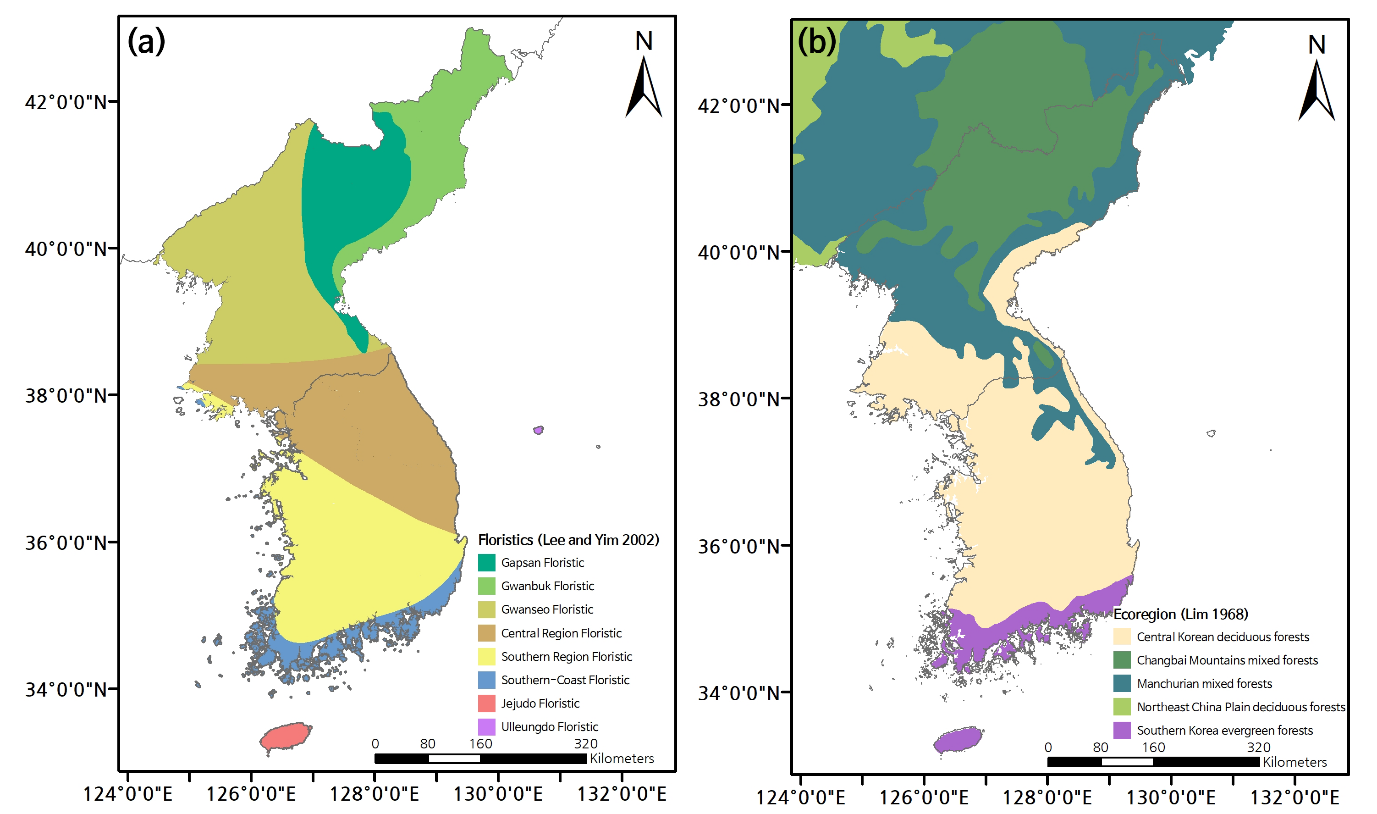


**Appendix S1. Maps showing the previously proposed floristic zones and ecoregions on the Korean Peninsula.** (a) Floristic zones (Lee and Yim, 2002); and (b) Ecoregion (Lim, 1968).


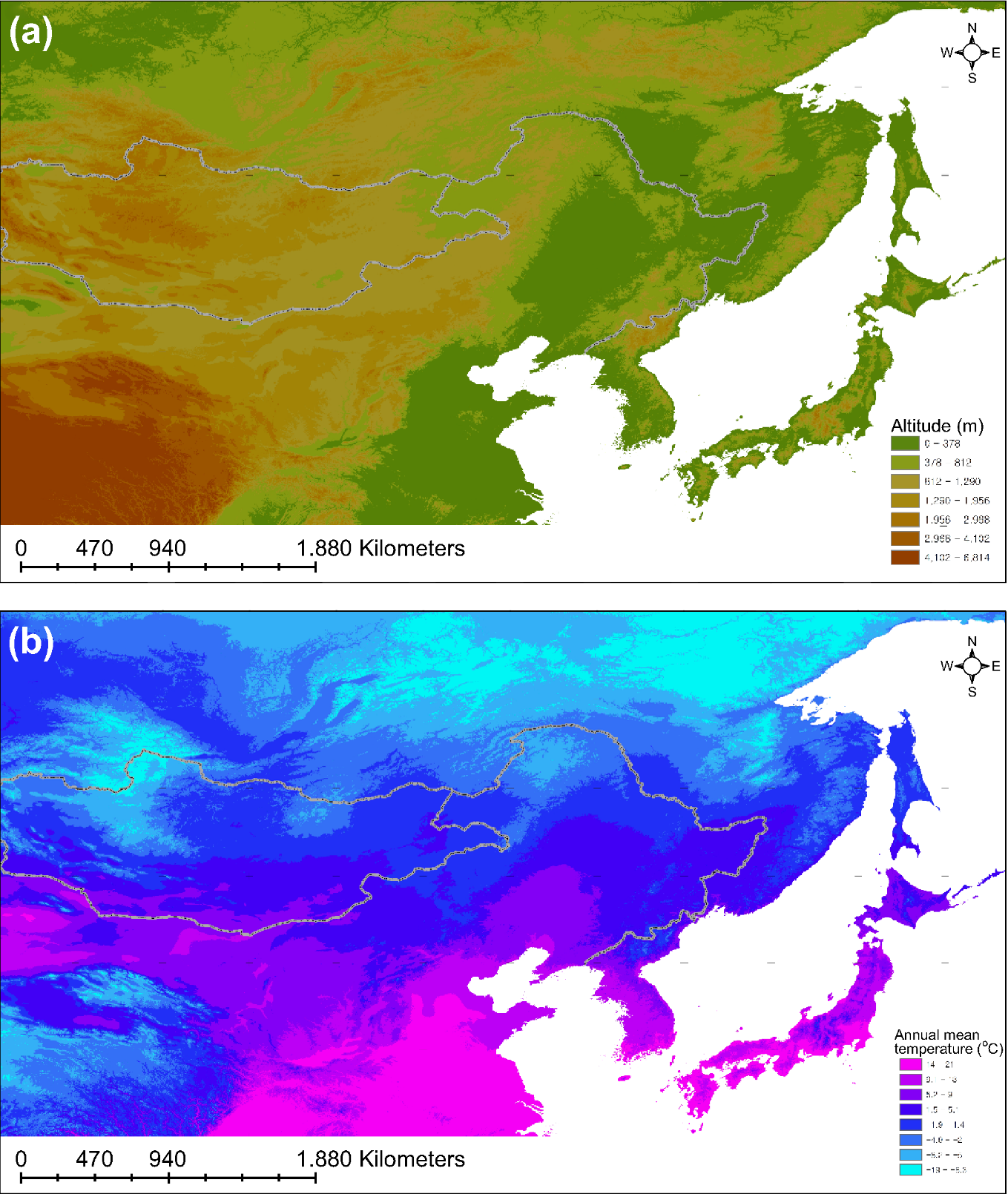


**Appendix S2. Map showing the altitude and mean annual air temperature in East Asia, including the Korean Peninsula.**


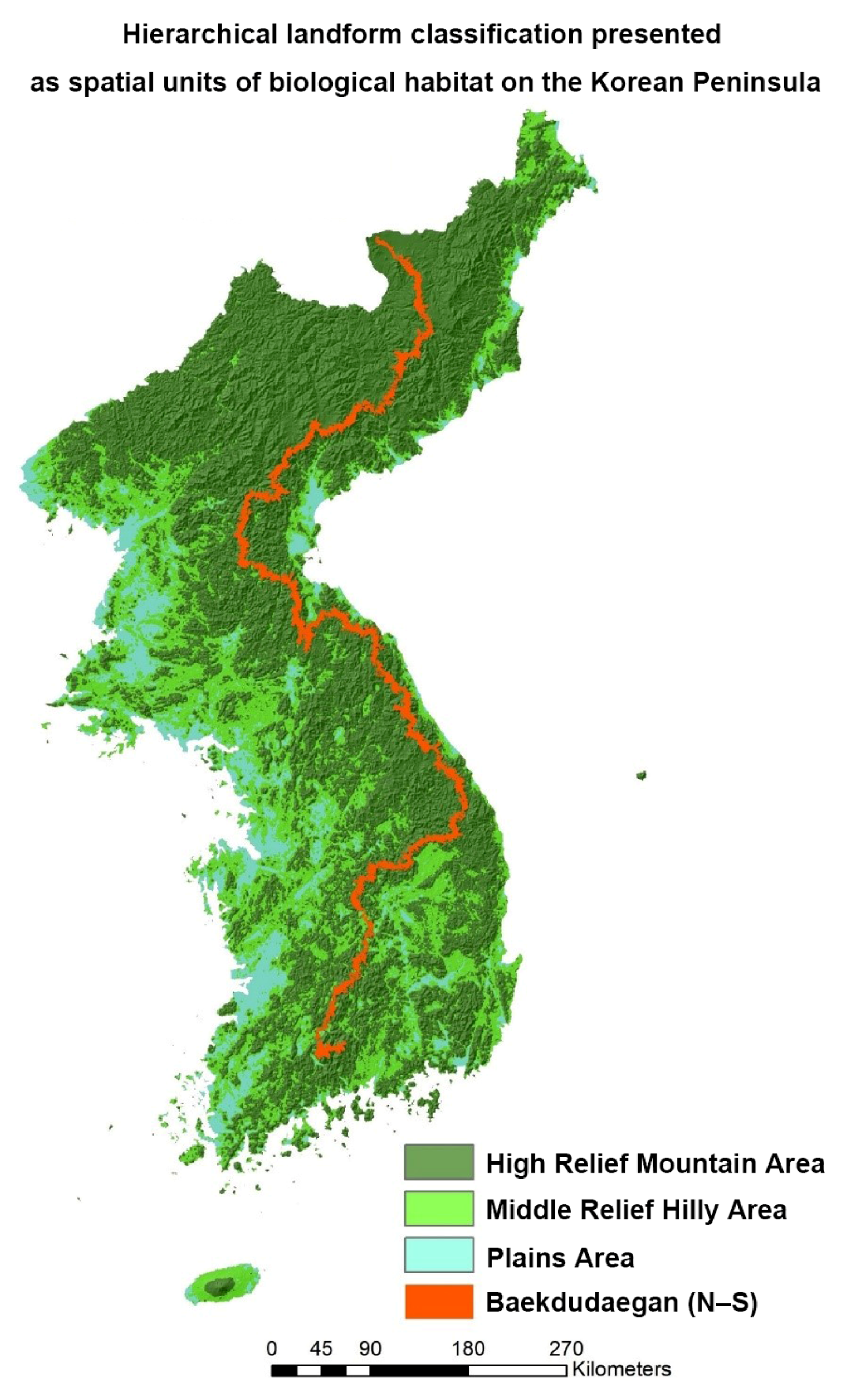


**Appendix S3. The hierarchical landform classification and the location of the Baekdudaegan mountain range on the Korean Peninsula.**


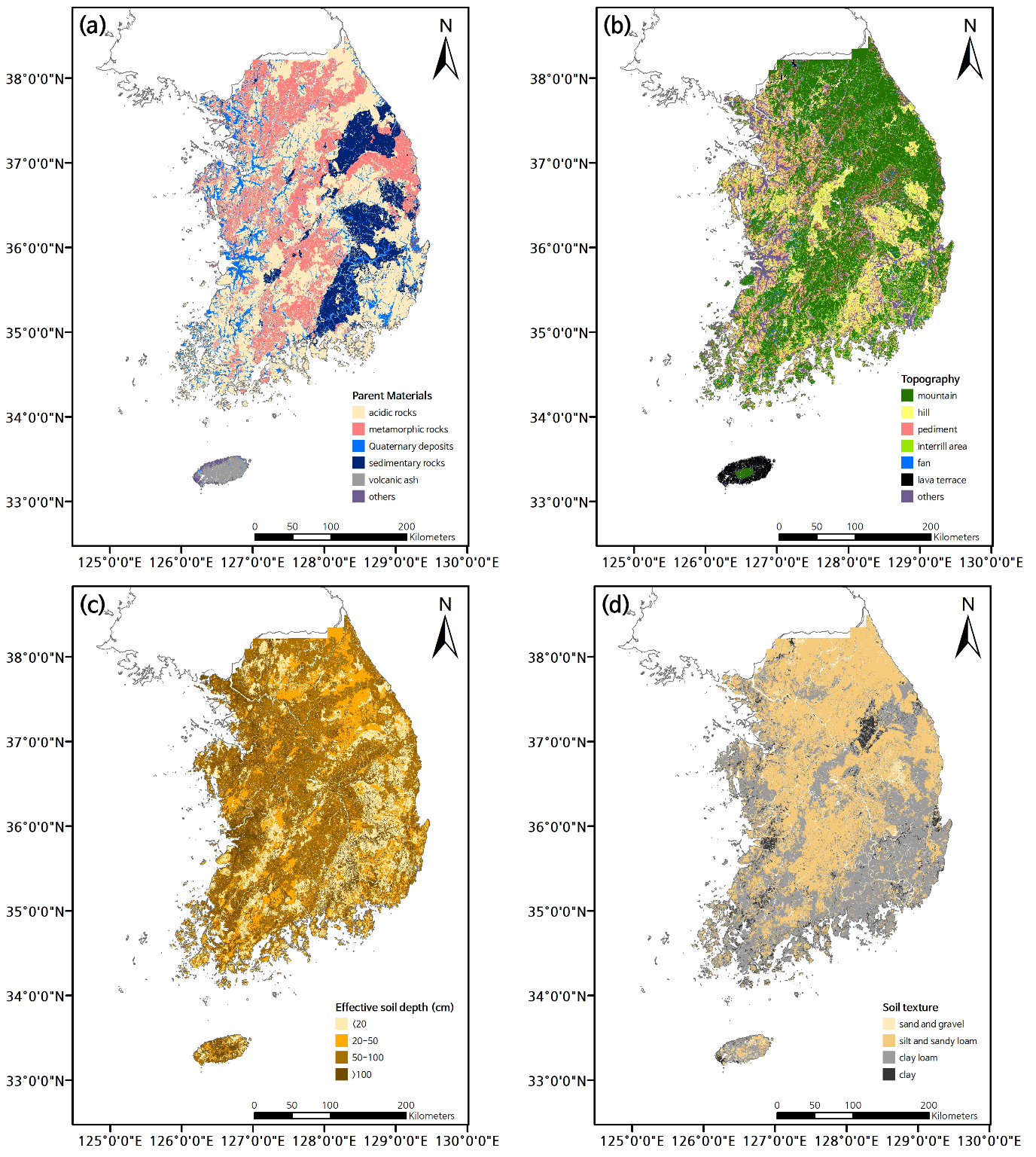


**Appendix S4. Maps showing the distribution of (a) parent materials, (b) topography, (c) effective soil depth, and (d) soil texture on the Korean Peninsula.**
